# The Rationale for a Preventative HCV Virus-Like Particle (VLP) Vaccine

**DOI:** 10.3389/fmicb.2017.02163

**Published:** 2017-11-07

**Authors:** Joseph Torresi

**Affiliations:** Department of Microbiology and Immunology, The Peter Doherty Institute for Infection and Immunity, University of Melbourne, Parkville, VIC, Australia

**Keywords:** Hepatitis C, neutralizing antibody, virus like particles, Hepatitis C vaccines

## Abstract

HCV represents a global health problem with ~200 million individuals currently infected, worldwide. With the high cost of antiviral therapies, the global burden of chronic hepatitis C infection (CHCV) infection will be substantially reduced by the development of an effective vaccine for HCV. The field of HCV vaccines is generally divided into proponents of strategies to induce neutralizing antibodies (NAb) and those who propose to elicit cell mediated immunity (CMI). However, for a hepatitis C virus (HCV) vaccine to be effective in preventing infection, it must be capable of generating cross-reactive CD4+, CD8+ T cell, and NAb responses that will cover the major viral genotypes. Simulation models of hepatitis C have predicted that a vaccine of even modest efficacy and coverage will significantly reduce the incidence of hepatitis C. A HCV virus like particle (VLP) based vaccine would fulfill the requirement of delivering critical conformational neutralizing epitopes in addition to providing HCV specific CD4^+^ and CD8^+^ epitopes. Several approaches have been reported including insect cell-derived genotype 1b HCV VLPs; a human liver-derived quadrivalent genotype 1a, 1b, 2, and 3a vaccine; a genotype 1a HCV E1 and E2 glycoprotein/MLV Gag pseudotype VLP vaccine; and chimeric HBs-HCV VLP vaccines. All to result in the production of cross-NAb and/or T cell responses against HCV. This paper summarizes the evidence supporting the development of a HCV VLP based vaccine.

## Introduction

The substantial global health burden posed by hepatitis C infection can only be addressed in part by antiviral therapies, regardless of their success rates. New and reinfection of high risk populations, such as people who inject drugs (PWID) (Sacks-Davis et al., 2013) makes the expectation of controlling hepatitis C infection with antiviral drugs alone unrealistic (Sievert et al., 2014). In Australia, there is an estimated 308,110 persons infected with hepatitis C virus (HCV), with a prevalence of HCV of 1.3%. It is estimated that 80,000 individuals have cleared infection leaving a total number of viraemic cases of 230,000 (prevalence of 1.0%). The problem of hepatitis C in Australia is also compounded by the fact there are an estimated 50,000 individuals who are unaware that they are infected. This means that there is a large residual pool of chronically infected individuals who will contribute to the ongoing transmission of HCV (Sievert et al., 2014). A similar picture exists in the US where there are ~3.9 million infected individuals and an estimated 50% unaware of their diagnosis. The prevalence of HCV in the US is 1.67% (Rosenberg et al., 2017). China, also has a large burden of HCV with an estimated 25–50 million infected individuals and a prevalence of 1.8–3.7% of the populations, accounting for 15–30% of all HCV infected individuals globally (Sievert et al., 2011; Peng et al., 2015; Wu et al., 2017; Zhang et al., 2017).

Limited access to treatment has also hampered the control of HCV. Several modeling studies have shown that for antiviral therapies alone to control HCV the uptake of treatment will need to increase substantially (Hellard et al., 2012; Martin et al., 2013; Sievert et al., 2014). A recent Australian study examined three treatment scenarios based on the availability of directly acting antiviral agents (Sievert et al., 2014). The outcomes of this study could be extrapolated to similar populations in the US and Europe. In the first scenario the effect of more efficacious treatments alone on HCV burden was assessed. This resulted in a fall in the predicted number of chronically infected individuals by 12,300 over the first 18 years of the introduction of these treatments. HCV-related mortality, the prevalence of compensated and decompensated cirrhosis and hepatocellular carcinoma (HCC) were reduced by 4%, respectively. In a second scenario, the impact of both highly effective therapies together with an expanded uptake of these treatments was assessed. This approach was predicted to be far more effective, resulting in a 60% reduction in the predicted number of chronically infected individuals. This was associated with a 43% reduction in mortality associated with HCV and a 52% reduction in both compensated and decompensated cirrhosis. The number of cases of HCC was also predicted to be 45% lower. These reductions were associated with a 24% reduction in the overall cumulative cost of treating CHCV. The last scenario examined the predicted impact of implementing highly effective therapies only in individuals with advanced fibrosis in the first 3 years followed by broadening access to these treatments to all individuals with CHCV. Of the three treatment scenarios this proved to be the most effective, with a 56% reduction in the predicted number of individuals with CHCV. This was associated with a 52% reduction in the mortality associated with CHCV, a 56 and 54% fall in the number of cases of compensated and decompensated cirrhosis, respectively, and halving of the number of cases of HCC. However, these reductions were only associated with a 26% reduction in the cumulative costs of managing CHCV (Sievert et al., 2014).

In another study, the impact of scaling up antiviral treatment was estimated in three cities with increasing HCV prevalence rates in populations of people who inject drugs (PWID), Edinburgh (baseline prevalence 25%), Melbourne (baseline prevalence 50%), and Vancouver (baseline prevalence 65%). Increasing treatment rates from around 5/1,000 PWID annually to 80/1,000 PWID annually over a 15-year period resulted in an estimated 90% reduction in HCV prevalence in Edinburgh and Melbourne but only an approximate 55% reduction in Vancouver (Martin et al., 2013). These findings suggest that especially in a high prevalence population, even a 20-fold increase in treatment rates would be insufficient to eliminate HCV.

Reinfection after treatment also remains a problem in a proportion of individuals. Some studies examining HCV reinfection in PWID who have cleared infection with antiviral therapy have suggested that the rates of reinfection are low. However, the follow-up period in these reports was relatively short and possibly insufficient to determine the true rate of reinfection (Grebely et al., 2010; Grady et al., 2012). In contrast studies that have examined a longer-term follow period up have reported high rates of reinfection, with one study from Australia reporting a high prevalence of late recurrence of viremia and reinfection of 17% (Bate et al., 2010). A study from Norway also examined reinfection after a sustained virological response (SVR) to antiviral therapy and followed patients for over 8 years (Midgard et al., 2016). Of individuals who had a history of injecting drug use 11% became reinfected with HCV. In contrast, in those who returned to injecting drug use 27% became reinfected with HCV. Interestingly the majority of reinfections occurred 5–8 years after having achieved a SVR with antiviral therapy (Midgard et al., 2016). These studies highlight that reinfection with HCV is not an infrequent occurrence, even after successful antiviral therapy.

## Modeling the impact of HCV vaccines

Limitations in access to antiviral therapies for HCV for all chronically infected individuals and the limited ability to significantly scale up access to treatment globally along with significant reinfection rates after clearance of HCV will negatively impact on our ability to eliminate hepatitis C infection and disease. An effective vaccine would be a substantial benefit to the overall aim of eradicating HCV globally. By utilizing mathematical models of HCV infection in high risk populations, it has also been possible to demonstrate that the introduction of a preventative HCV vaccine would have a substantial benefit in reducing the incidence of CHCV (Hahn et al., 2009; Scott et al., 2015).

One such study from San Francisco examined three vaccination strategies (Sacks-Davis et al., 2013); no targeting and selecting PWID for vaccination at random; risk targeting which prioritized high-risk PWID; and sero-targeting, which prioritized HCV antibody negative individuals to receive vaccine (Hahn et al., 2009). The investigators also modeled predicted vaccine efficacies of 50, 65, and 80% and vaccination coverage rates of 0.2, 0.6, and 1% PWID per month. In an approach targeting high-risk individuals or by sero-targeting, the incidence of CHCV was predicted to fall 13.5–4.3, 3.2, and 2.3% per person years at 5, 10, and 30 years after the introduction of a vaccine with 80% efficacy and with a high vaccination rate of 15% per month. The predicted reduction in HCV incidence was only marginally lower if the vaccination coverage fell to 0.6% per month, resulting in a fall in the incidence of CHCV to 2.9% after 30 years. Finally, even a vaccine with 50% efficacy and a moderate to high coverage rate targeted to high-risk PWID was predicted to dramatically reduce the incidence of HCV at 30 years (Hahn et al., 2009).

A more recent UK study also showed that a halving chronic HCV prevalence was possible over a period of 40 years by achieving annual vaccination rates of 162 (72%), 77 (56%), and 44 (38%) per 1,000 PWID for low (50% protection for 5 years), moderate (70% protection for 10 years), and high (90% protection for 20 years) vaccine efficacies. The model predicted that HCV prevalence could be halved over 20 years and this required a doubling in vaccination rates, but only for vaccines of moderate and high efficacy (Stone et al., 2016).

Finally, a subsequent simulation study has shown that vaccination after successful treatment with DAAs is also be as effective at reducing HCV prevalence as vaccinating an equivalent number of people who inject drugs (PWID) in the community (Scott et al., 2015). In this study, a vaccine of 60–90% efficacy was predicted to be beneficial in reducing HCV prevalence especially in populations of PWID with high (50%) to very high (75%) initial chronic HCV prevalence (Scott et al., 2015).

## The importance of neutralizing antibody responses in protection and viral clearance

The clearance of hepatitis C infection requires strong and broad cross-reactive T cell and neutralizing antibody (NAb) responses.

Although the proportion of individuals is lower and the time to clearance of HCV after reinfection is twice as likely compared to individuals with primary infection (Sacks-Davis et al., 2013) this does not necessarily result in complete protection against reinfection, suggesting that sterilizing immunity against HCV may not occur frequently (Aitken et al., 2008; Osburn et al., 2010; Grebely et al., 2012; Sacks-Davis et al., 2013). However, these findings indicate that the development of a vaccine that is able to produce protective immunity to prevent persistent infection is possible. The development of early and multi-specific class 1 CD8+ and class II CD4+ T cell and NAb responses during acute HCV infection are associated with the spontaneous clearance of infection (Schulze Zur Wiesch et al., 2012). T cell responses to HCV have been reviewed comprehensively elsewhere (Park and Rehermann, 2014; Holz and Rehermann, 2015). However, the clearance of HCV may require substantially more than strong T cell responses alone (Puig et al., 2006). There is strong evidence that cross-NAb responses to epitopes in the E2 glycoproteins are associated with HCV clearance of existing infection and protection against new HCV infection in animal models.

In humans, the early induction of NAb has been associated with control of viraemia and resolution of infection (Pestka et al., 2007). Similarly, immunoglobulin prepared from the serum of anti-HCV positive humans (Yu et al., 2004) protects chimpanzees against viral challenge. In this study, immune globulin samples prepared from the anti-HCV positive donor plasma were first tested for HCV neutralizing activity using an *in vitro* infectious HCV pseudoparticle assay. The IgG showing neutralizing activity in this assay were then mixed with a standardized HCV inoculum prepared from the acute phase plasma of a patient with hepatitis C infection before been administered to chimpanzees. Control chimpanzees were administered the same inoculum mixed with HCV Ab negative IgG. Weekly serum samples were collected from the chimpanzees and tested for the presence of HCV viremia using RT-PCR. The chimpanzees administered with the mixture of HCV neutralizing IgG and inoculum were protected against HCV infection while the control chimpanzees became infected (Yu et al., 2004).

The early appearance of broad NAb is also an important determinant of clearance of HCV and it is the breadth and not just the presence of heterologous NAb alone that has been associated with protection against persistent infection with HCV (Osburn et al., 2014).

Early studies in chimpanzees has provided valuable insight into the importance of NAb to HCV. These studies demonstrated that NAb develops against the hypervariable region 1 (HVR1) of the E2 glycoprotein and that neutralization of HCV with anti-HVR1 antibodies prevents HCV infection in chimpanzees (Farci et al., 1994, 1996). In addition, vaccination of chimpanzees with mammalian cell expressed recombinant E1 and E2 glycoproteins resulted in protection against challenge with HCV (Choo et al., 1994; Rosa et al., 1996). The recombinant E1 and E2 proteins were produced in HeLa or CHO cells and purified by affinity chromatography and ultrafiltration. Chimpanzees were injected recombinant HCV antigens and received several immunizations over a 7–9-month period followed by challenge with HCV-1 isolated from a chronically infected chimpanzee. None of the immunized chimpanzees became viremic following challenge in contrast to the control animals, all of which became infected (Choo et al., 1994). In a subsequent study the ability of antibodies from the immunized chimpanzees to neutralize the binding of recombinant E2 to MOLT-4 cells was assessed (Rosa et al., 1996). In this report, the neutralization of binding of HCV E2 protein to MOLT-4 cells was found to correlate with protection against challenge with HCV-1.

Neutralization of HCV with rabbit hyperimmune serum to a homologous HVR1 sequence prior to injection into chimpanzees has also been demonstrated (Farci et al., 1994). Following on from these studies it was shown that immunoglobulin prepared from the serum of anti-HCV positive humans protected chimpanzees against viral challenge (Yu et al., 2004). In addition, cross-NAb to epitopes in the E1 and E2 glycoproteins of HCV have also been shown to be protective (Owsianka et al., 2008; Broering et al., 2009; Dowd et al., 2009; Raghuraman et al., 2012).

The limited number of suitable animal models has hampered ongoing studies into the nature of protective NAb responses to HCV. However, the development of humanized uPa-SCID liver chimeric mice has enabled significant progress to be made (Meuleman et al., 2008). Using this model it has been possible to demonstrate that human IgG from HCV-infected individuals and human anti-E2 monoclonal antibodies protect uPA-SCID liver chimeric mice against homologous and heterologous HCV challenge (Law et al., 2008; Vanwolleghem et al., 2008; Meuleman et al., 2011). Furthermore, broad NAb are able to cure uPA-SCID liver chimeric mice of HCV infection (de Jong et al., 2014). In a separate model, anti-E2 sero-positive immunocompetent humanized mice were protected against homologous HCV challenge with a recombinant vaccinia-HCV virus encoding the structural proteins of HCV (Dorner et al., 2011). Furthermore, broad neutralizing human monoclonal antibodies that interfere with the binding of HCV E2 to CD81 on the surface of hepatocytes and a second subset of broad NAbs recognizing native E1E2 heterodimers protect liver chimeric mice against heterologous HCV challenge (Giang et al., 2012). These studies all reinforce the important role of NAb in protection against hepatitis C infection (Owsianka et al., 2008; Broering et al., 2009; Dowd et al., 2009; Raghuraman et al., 2012). Although, studies in liver chimeric mice have provided valuable information regarding NAb to HCV they are immunocompromised and therefore limited by the inability to directly assess the immunogenicity of HCV vaccines.

## HCV NAb vaccine approaches

A number of approaches have been developed to induce the production of broad protective NAb responses. It has been possible to prevent persistent infection with both homologous and heterologous HCV challenge in chimpanzees following vaccination with recombinant E1 and E2 glycoproteins produced in mammalian cells. Recombinant HCV E1E2 adjuvanted with MF59C is also safe and immunogenic in humans resulting in the production of NAb and CD4^+^ T cell responses (Frey et al., 2010). Furthermore, immunization of human volunteers with recombinant gpE1/E2 (HCV genotype 1a) resulted in the production of broad cross-NAb responses (Law et al., 2013). Finally, IgG isolated from mice immunized with inactivated cell culture derived HCV has been shown to neutralize HCV pseudo-particles and protect liver chimeric mice against homologous HCV challenge (Akazawa et al., 2013).

Although, a vaccine derived from a single genotype can elicit NAb, the inclusion of antigens of a number of different genotypes is expected to produce a broader cross-NAb response and thereby be more effective than a single antigen. Broad NAbs act by recognizing native E1E2 heterodimers (Giang et al., 2012) and by blocking the interaction of the virus with critical co-receptors like CD81, scavenger receptor class B type 1 (SR-B1) and Claudin-1 (Scarselli et al., 2002; Koutsoudakis et al., 2007; Zeisel et al., 2007; Meuleman et al., 2008; Douam et al., 2014). For a vaccine to be able to produce effective NAb responses it is therefore likely that such a vaccine will need to express native E1E2 structures. The significance of these regions on the viral envelope is further highlighted by the ability of polyclonal and monoclonal antibodies directed to these regions to protect human liver chimeric mice against HCV challenge (Meuleman et al., 2011). These findings are highly significant for HCV vaccine design because they reinforce the need to present both E1 and E2 proteins in the correct structural organization in a vaccine.

## Hepatitis C virus-like particles (HCV VLPs) as neutralizing vaccine candidates

A number of HCV vaccines that produce cellular immune responses against the core or non-structural proteins have entered clinical trials. A recent Phase I study reported using chimpanzee Ad3 and MVA encoding the HCV non-structural (NS) proteins in a prime-boost approach (Swadling et al., 2014). The adenoviral and MVA HCV vaccine were safe and resulted in the production of strong cross-genotypic CD4+ and CD8+ T cell responses. However, in a meta-analysis of the efficacy of various vaccine approaches in chimpanzees it was found that protection against HCV was more closely correlated with immune responses against the viral structural proteins rather than the non-structural proteins (Dahari et al., 2010). An effective preventative vaccine will therefore need to include HCV structural in addition to non-structural proteins in order to produce broad T and B cell responses. HCV VLPs have the potential to fulfill these requirements (Table 1).

**Table 1:** Summary of various HCV VLP approaches and immune responses reported in different animal models.

**HCV VLP type**	**Genotype**	***In-vitro* cell line for production**	**Neutralization HCVcc or HCVpp (Genotype)**	**Binding of human NAb**	**DC maturation**	**Memory B cell responses**	**HCV specific CD8+ and/or CD4+ T cell responses**	**Animal models tested**	**Protection from HCV Challenge**	**References**
Insect cell derived	1b	Sf9	No Strong antibody response in mice. Neutralization of vaccinia virus-HCV with Baboon immune sera. (Poor antibody responses in Chimpanzees)	Yes	Yes (*in-vitro*)	Yes	Yes (CD4+, CD8+, CTL)	(1) BALB/c mice (2) HLA2.1 transgenic mice (3) Baboons (4) Chimpanzee	(1) BALB/c and HLA2.1 transgenic mice vaccinia virus-HCV (2) Protection against persistent infection with homologous HCV in Chimpanzees	Baumert et al., 1998, 1999; Murata et al., 2003; Steinmann et al., 2004
Mammalian cell derived	1a, 1b, 2, 3a	Huh7	Yes (HCVcc 1b and 2)	Yes	Yes (*in-vitro*)	Yes	Yes (CD4+, CD8+, Granzyme B)	(1) C57BL/6 and BALB/c mice, (2) HLA2.1 transgenic mice	No	Chua et al., 2012; Earnest-Silveira et al., 2016b
**CHIMERIC HBsAg/HCV LPs**										
(1) HBs-HCV E1 and E2 VLPs	1a	CHO	Yes (HCVcc and HCVpp 1a, 1b, 2a, and 3)	No	No	No	No	Rabbits	No	Patient et al., 2009; Beaumont et al., 2013
(2) HBsAg/HCV HVR1	1a and 1b	Huh7	Yes (HCVpp 1a)	No	No	No	No	C57BL/6 and BALB/c mice	No	Netter et al., 2001, 2003; Woo et al., 2006; Haqshenas et al., 2007; Vietheer et al., 2007; Cheong et al., 2009
**RETROVIRAL HCV-LIKE PARTICLES**										
(1) RetroVLP^E1E2^ (2) RetroVLP^NS3^ (3) Plasmo-retroVLP^E1E2^ (4) rAdE1E2 (Prime-boost strategies)	1a		Yes (1b, 2a, 2b, 4, and 5)	No	No	No	Yes (CD8+, CTL)	(1)C57BL/6 and BALB/c mice (2) Macaques	No	Bartosch et al., 2003; Bellier et al., 2006, 2009; Desjardins et al., 2009; Garrone et al., 2011; Huret et al., 2013

HCV VLPs elicit both NAb and cellular immune responses (Chua et al., 2012; Beaumont et al., 2013; Kumar et al., 2015; Earnest-Silveira et al., 2016a; Table 1). Compared to HCV core, E1 and E2 DNA vaccines HCV VLPs produce stronger cytotoxic T cell responses in mice (Murata et al., 2003). This is significant given the important role of HCV core specific T cell responses in clearance of HCV (Smyk-Pearson et al., 2006). HCV VLPs produced in insect cells also produce maturation of dendritic cells (DC) (Barth et al., 2005; Chua et al., 2012; Earnest-Silveira et al., 2016b), another important consideration given the role of DC in directing adaptive immune responses to viruses and vaccines (Li et al., 2006; Yu et al., 2008; Alloatti et al., 2016). These are important findings for the development of HCV VLP vaccine as both the human papilloma virus (HPV) and hepatitis B virus (HBV) VLP vaccines have been shown to result in strong T cell responses in humans (Pinto et al., 2006; Vandepapeliere et al., 2008; Yokomine et al., 2017).

The development of cross-NAb to epitopes on the surface of HCV that develop in the course of natural infection provides further encouragement for the development of a neutralizing HCV VLP vaccine (Mancini et al., 2009). Furthermore, as HCV-specific NAbs recognize tertiary or quaternary structures (Giang et al., 2012) this makes VLPs attractive as a potential vaccine for HCV (Garrone et al., 2011; Chua et al., 2012; Beaumont et al., 2013). HCV VLPs are able to present conformational epitopes in their native state and consequently several approaches have been described. Favorable immune responses, both humoral and cellular, have been shown with an insect cell-derived vaccine based on genotype 1b HCV VLP. This vaccine was also partially protective in chimpanzees (Elmowalid et al., 2007). A HCV genotype 1a E2 glycoprotein/retroviral Gag pseudotype vaccine has also been shown to produce high-titre NAb in both mice and macaques (Garrone et al., 2011). Vaccination of rabbits with chimeric HBV-HCV VLPs produced in CHO cells and containing genotype 1a E1E2 heterodimers has also been shown to result in the production of cross-NAb responses against heterologous HCV genotypes (Beaumont et al., 2013; Table 1).

### Neutralizing epitopes in the E2 protein

The E2 protein contains the major conformational neutralizing antigenic regions organized into a number of important antigenic domains (A–E or the AR3 and AR4 regions; Keck et al., 2004, 2008; Giang et al., 2012; Pierce et al., 2016). Antibodies against these epitopes have been shown to be broadly cross-neutralizing and protective against HCV challenge in human liver chimeric mice (Giang et al., 2012).

**Figure 1:**
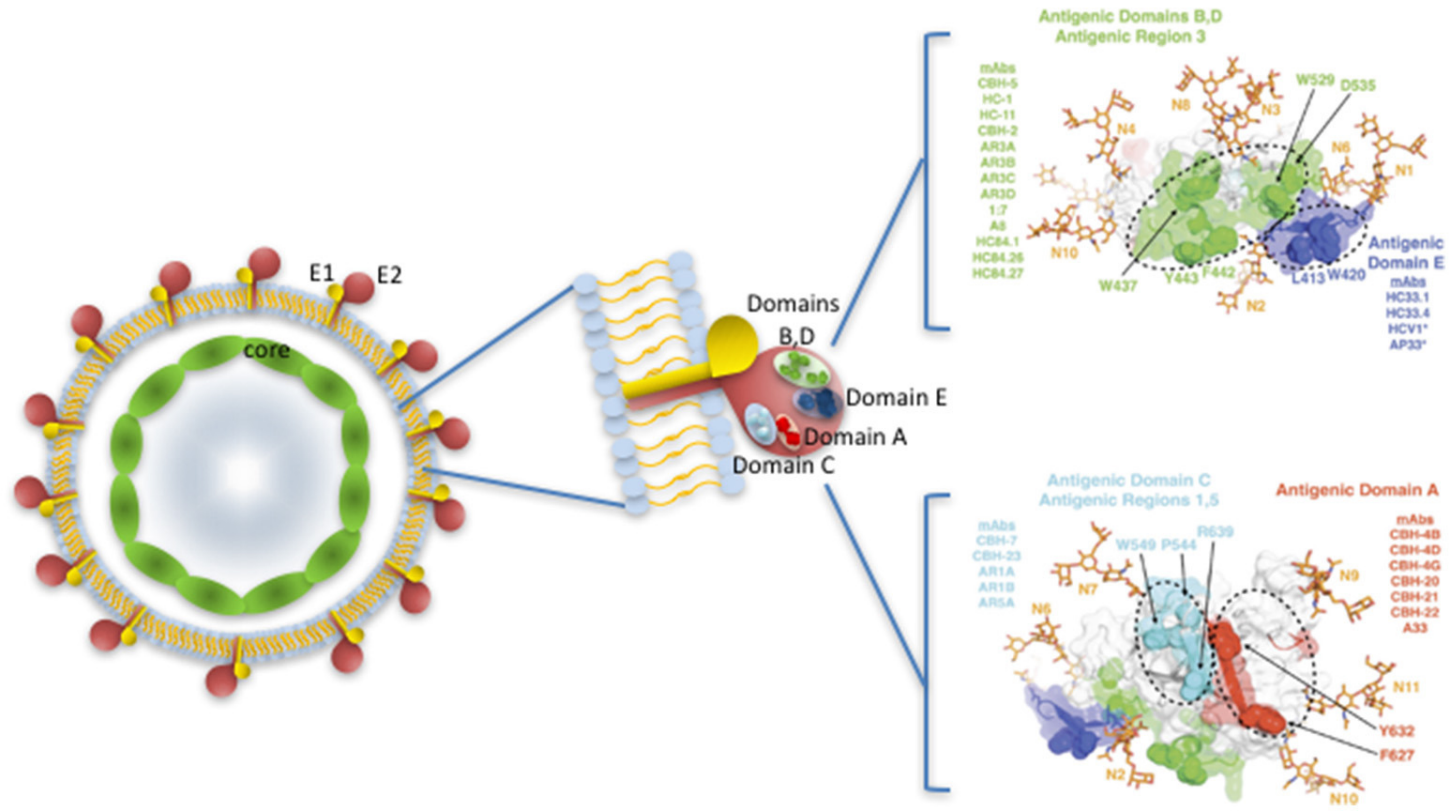
HCV virus like particle showing E1 and E2 protein dimers embedded in a lipid bilayer, surrounding the core nucleocapsid. Major neutralizing regions are situated on the surface of the E2 protein. Antigenic domains and antigenic regions with the key binding sites of key human Mabs are shown on the right, with the neutralizing antibody face (antigenic domains B, D; Antigenic Region 3; antigenic domain E) above and the non-neutralizing face (antigenic domains A,C) of E2 below. Adapted from Fauvelle et al. (2016) and with permission from Prof S Foung.

**Figure 2:**
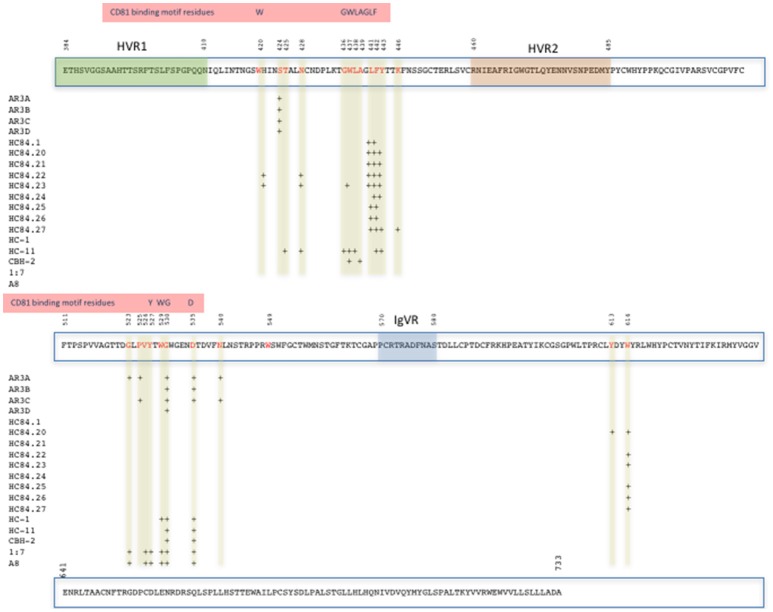
Map of the E2 protein showing the position of amino acid contact residues (red) of HuMabs binding the Domains B and D and Antigenic Region 3 on the neutralizing face of E2 (Pierce et al., 2016). The positions of HVR1, HVR2, IgVR, and the CD81 binding motif residues on E2 are highlighted.

Distinct broad neutralizing epitopes in and downstream of hypervariable region 1 (HVR1) of the E2 protein have been described (Keck et al., 2013, 2014), with at least four distinct clusters of overlapping epitopes mediating broad virus neutralization (Keck et al., 2004, 2008, 2011). It has also been possible to define a neutralizing and a non-neutralizing face to the E2 core antigenic surface (Kong et al., 2013; Khan et al., 2014). Together with comprehensive alanine substitution studies it has also been possible to map the binding of human monoclonal antibodies (HuMAbs) to these critical regions of E2, with the most potent binding to domains B and D of E2 the protein (Fauvelle et al., 2016). Several HuMabs also bind to the non-neutralizing face of E2 (Fauvelle et al., 2016; Figure 1). In a recent report, a comprehensive analysis of the E2 binding residues of neutralizing HuMabs was described (Pierce et al., 2016). This study identified the critical binding residues for HuMAbs on the neutralizing face of E2 and for which limited epitope mapping was previously described (Figure 2) together with the critical residues defining the major antigenic domains A–D of the E2 protein. It also mapped the binding residues for HuMAbs targeting antigenic domains A and C for which there was little or no previous epitope mapping data nor crystal structures available (Pierce et al., 2016; Figure 1). This information provides critical data for the design of neutralizing antibody vaccines for HCV.

### Neutralizing epitopes in the E1 protein

The E2 glycoprotein also contains neutralizing epitopes, although there is less information available characterizing these. A broad cross-neutralizing epitope has been identified between amino acids 313 and 327 (Meunier et al., 2008). Monoclonal antibodies to these epitopes neutralized HCV/retroviral pseudotype particles (HCVpp) of genotypes 1a, 1b, 4a, and 6a and HCVcc of genotype 1a and 2a. By using epitope extraction and mass spectrometry it has also been possible to identify a potentially neutralizing epitope in E1 (aa 313 and 327) that overlaps the cross-neutralizing epitope 313–327 described by Meunier and coworkers (Grollo et al., 2006; Torresi et al., 2007; Meunier et al., 2008). A more recent study using chimeric hepatitis B S envelope protein subviral particles expressing HCV E1 and E2 proteins alone or as E1E2 dimers also demonstrated that anti-E1 antibodies produced following immunization of rabbits were able to neutralize HCVcc *in vitro* and had additive cross neutralizing activity with anti-E2 antibodies against heterologous HCVcc genotypes *in vitro* (Beaumont et al., 2016). This study reinforces the importance of including both E1 and E2 in a neutralizing HCV vaccine.

These studies highlight the complex organization of neutralizing epitopes on the surface of HCV and argue that for a vaccine to be effective it would need to be able to closely reproduce these quaternary structures (Pierce et al., 2016). An HCV VLP based vaccine will fulfill the requirement of delivering critical conformational neutralizing epitopes in addition to providing HCV core specific CD4+ and CD8+ epitopes.

## Insect cell derived HCV VLPs

Insect cell-derived VLPs produced using a baculovirus system expressing the core, E1 and E2 structural proteins of HCV (genotype 1b) were able to induce broad HCV specific immune responses (Baumert et al., 1998, 1999; Murata et al., 2003; Steinmann et al., 2004). These studies first demonstrated that the HCV structural proteins were able to self-assemble into 40–60 nm VLPs that were representative of HCV (Baumert et al., 1998). The HCV VLPs were shown to be similar structurally and antigenically in composition to putative HCV virions (Baumert et al., 1999). However, the glycosylated forms of E1 and E2 and the size of E2 in the insect cell derived HCV VLPs was different from E1 and E2 expressed in mammalian cells and this was attributed in part to the less efficient cleavage of E2 in insect cells (Baumert et al., 1999). Immunization of mice resulted in antibody responses against the HCV structural proteins and the HCV VLPs were immunoreactive with sera from individuals infected with different HCV genotypes suggesting that the HCV VLPs may have exhibited broad cross-neutralizing epitopes (Baumert et al., 1998). In a follow up study, HLA-A2.1 transgenic mice were immunized with genotype 1b HCV VLPs. Humoral responses were assessed by ELISA while T cell responses were assessed using pools of HCV core and envelope specific peptides in ELISPOT and intracellular cytokine staining assays (Baumert et al., 1998, 1999; Murata et al., 2003; Steinmann et al., 2004). The HCV VLP immunized mice developed strong humoral and HCV core-specific CD4+ and CD8+ T cell responses (Baumert et al., 1998, 1999; Murata et al., 2003; Steinmann et al., 2004). In a surrogate HCV challenge model to assess the importance of these immune responses, BALB/c and HLA-A2.1 mice were immunized with the vaccine and then challenged with recombinant vaccinia virus expressing the HCV structural proteins (vvHCV.S) and compared to unimmunized mice as a negative control followed by harvesting of the mouse ovaries and determining vaccinia virus titres by plaque assay. In contrast to negative control mice, the HCV VLP immunized mice had >5 log10 reductions in viral titres and no detectable viremia. Finally, adoptive transfer of splenocytes from immunized BALB/c mice protected non-immunized mice from vvHCV.S challenge (Baumert et al., 1998, 1999; Murata et al., 2003; Steinmann et al., 2004). In addition, the HCV VLPs were able to stimulate the maturation of human dendritic cells (Barth et al., 2005), an important response for an effective vaccine. The HCV VLPs demonstrated a saturable binding to human hepatoma cells that was efficiently blocked by anti-E2 antibodies (Wellnitz et al., 2002). Antibodies and IgG isolated from the sera of patients with acute and chronic hepatitis C infection also inhibited the binding of HCV VLPs to human hepatoma cells (Steinmann et al., 2004).

Immunization of HLA2.1 transgenic mice with the insect cell-derived HCV VLPs also resulted in the production of strong humoral and HCV-specific T helper and CTL responses (Lechmann et al., 2001). The HCV-specific T cell responses produced in the HLA2.1 transgenic mice were also substantially stronger than responses against recombinant HCV protein and DNA based vaccines (Lechmann et al., 2001; Murata et al., 2003). In a surrogate challenge model, immunized mice (including HLA2.1 transgenic mice) were protected from challenge with recombinant vaccinia virus expressing HCV structural proteins (Murata et al., 2003). The significant role of both CD4+ and CD8+ T cells in providing protection against vaccinia-HCV was confirmed with adoptive transfer experiments following lymphocyte depletion (Murata et al., 2003).

The immunogenicity of HCV VLPs and the effects of novel adjuvants were further tested in a nonhuman primate model (Jeong et al., 2004). Baboons were immunized with four doses of HCV VLPs plus adjuvant AS01B alone or the combination of AS01B and CpG oligodeoxynucleotides. All animals developed HCV specific antibody, CD4+ and CD8+ T cell responses. The addition of adjuvants did not significantly increase the immunogenicity of the HCV VLPs (Jeong et al., 2004).

These encouraging results paved the way to evaluate the immunogenicity of HCV VLPs in the chimpanzee (Elmowalid et al., 2007). Chimpanzees were immunized with HCV VLPs alone or with AS01B adjuvant. HCV-specific CD4+ and CD8+ T cell responses developed in all chimpanzees, although antibody responses were generally poor (Elmowalid et al., 2007). The chimpanzees were then challenged with HCV. One chimpanzee developed a transient low-level viremia. Three other chimpanzees developed high level viremia however all went on to clear infection 10 weeks after HCV challenge. The challenge with HCV also resulted in a significantly stronger peripheral and intra-hepatic T cell response. In contrast, 3 of 4 naïve chimpanzees developed persistent HCV infection. Vaccination of the chimpanzees was clearly able to induce immune responses that were able to control HCV and prevent progression to persistent infection (Elmowalid et al., 2007). The poor antibody responses to the vaccine were in contrast to the strong responses observed in mice and may have been a reflection of the production of the VLPs in insect cells. This is perhaps not surprising as E2 protein produced in insect cells has previously been shown to not to result in the production of protective antibody responses in primates (Choo et al., 1994; Rosa et al., 1996).

## Mammalian cell derived HCV VLPs

HCV VLPs of genotype 1a have also been developed in human hepatocyte-derived. These mammalian cell derived HCV VLPs produce NAb and HCV specific T cell responses in mice (Chua et al., 2012; Earnest-Silveira et al., 2016b). Similarly, a genotype 3a HCV VLP vaccine also induced broad humoral and cellular immune responses in mice (Kumar et al., 2015). HCV VLPs possess the biochemical and biophysical properties that are characteristic of HCV virions (Gastaminza et al., 2010; Catanese et al., 2013; Earnest-Silveira et al., 2016b). The envelope proteins E1 and E2 are produced as glycoprotein heterodimers that self-assemble with the HCV core protein into VLPs that are morphologically typical of HCV virions by electron microscopy (EM) and cryo-EM (Earnest-Silveira et al., 2016a,b). The morphology of these hepatocyte-derived HCV VLPs is consistent with the findings of other investigators (Gastaminza et al., 2010; Catanese et al., 2013) who have shown that HCV virions isolated from sera of infected individuals are spherical, heterogeneous, vary in size from 40 to 100 nm (Earnest-Silveira et al., 2016b). Like HCV virions, these HCV VLPs incorporate apolipoproteins C and E (Gastaminza et al., 2010; Catanese et al., 2013; Earnest-Silveira et al., 2016a). The entry of the genotype 1a VLPs into Huh7 cells could also be blocked effectively with IgG purified from the sera of individuals infected with HCV genotype 1a and 3a (Earnest-Silveira et al., 2016b). This shows that cross-NAb generated in the course of natural infection in humans are able to recognize potential neutralizing epitopes present on the surface of the HCV VLPs.

In a subsequent study, a genotype 3a HCV VLP vaccine was also shown to be strongly immunogenic (Kumar et al., 2015). The genotype 3a HCV VLP vaccine resulted in strong T cell and NAb responses in mice. In addition, a prime-boost strategy using a HCV DNA or recombinant adenovirus vaccine encoding genotype 3a HCV core, E1 and E2 proteins resulted in even stronger humoral (including NAb) and cellular immune responses (Kumar et al., 2015).

The mammalian cell derived HCV VLPs also induced the production of HCV-specific CD4+ and CD8+ T cell in mice. This is an important finding as spontaneous clearance of HCV, control of HCV viremia and the development of HCV specific memory B cell and NAb responses are all associated with the development of CD4^+^ T helper cell responses (Schulze Zur Wiesch et al., 2012; Raziorrouh et al., 2015; Spaan et al., 2015).

To advance this approach, a quadrivalent genotype 1a/1b/2a/3a HCV VLP vaccine was produced and a method for the large-scale production and refined purification of the vaccine to eliminate residual cellular proteins was established (Earnest-Silveira et al., 2016a). The immunogenicity of this vaccine in humanized liver chimeric mice and a large animal model and the binding of neutralizing human monoclonal antibodies (HuMAbs) targeting conserved antigenic domain B and D epitopes of the E2 protein (Keck et al., 2004, 2008, 2011, 2016; Fauvelle et al., 2016) to the HCV VLPs are currently under evaluation.

## Chimeric HBsAg/HCV LPs

HBsAg subviral particles have been used to deliver the complete envelope proteins of HCV (Patient et al., 2009; Beaumont et al., 2013, 2016; Beaumont and Roingeard, 2015) or specific E2 neutralizing epitopes (Netter et al., 2001, 2003; Woo et al., 2006; Vietheer et al., 2007). By replacing the transmembrane domain (TMD) of the HBV S protein a with the TMD of HCV genotype 1a E1 and E2 proteins it was possible to produce chimeric HBs-HCV particles expressing HBs protein fused to full-length E1 and E2 proteins (Patient et al., 2009). Chimeric particles were produced using either a recombinant Semliki forest virus expression system to transduce CHO cells or by producing stably transfected CHO cells. The chimeric fusion proteins efficiently self-assembled into VLPs containing HCV E1 and E2 glycoproteins (Patient et al., 2009; Beaumont et al., 2013). The immunogenicity of the chimeric HBs-HCV particles was assessed in rabbits and induced strong HCV E1 and E2 specific antibody responses against. Immune sera from rabbits vaccinated with chimeric HBs-HCV E1 or E2 particles neutralized both HCVpp and HCVcc of several genotypes, including 1a, 1b, 2a, and 3 (Beaumont et al., 2013). Antibody responses were also found to be less sustained in E1-S-immunized than in E2-S-immunized rabbits (Beaumont et al., 2013). The immunogenicity of the chimeric vaccine and the strength of the HCV E2 responses was not affected by pre-existing immunity to HBsAg (Beaumont and Roingeard, 2015). In a further follow-up study these investigators compared the immunogenicity and NAb responses of chimeric particles expressing HCV E1 and E2 proteins alone with chimeric particles expressing E1E2 heterodimers (Beaumont et al., 2016). Interestingly the investigators found that immunization of rabbits with the chimeric vaccine expressing the E1E2 dimers resulted in poorer NAb responses compared to the vaccine with E1 and E2 expressed separately. However, an additive effect was found in anti-E1 and anti-E2 responses that was associated with increased cross-neutralization of heterologous HCV genotypes (Beaumont et al., 2016). This finding reinforces the requirement of including both E1 and E2 proteins for an effective vaccine.

In an alternative approach Netter and coworkers modified HBsAg particles by introducing HCV specific B and T cell epitopes into the “a” determinant of the HBs protein (Netter et al., 2001, 2003; Woo et al., 2006; Haqshenas et al., 2007; Vietheer et al., 2007). Sequences from HVR1 of the E2 gene of HCV were inserted into the HBsAg gene so that varying lengths of the HCV HVR1 were expressed on the “a” determinant of the HBsAg protein (Netter et al., 2001). By using this approach it was possible to produce HBsAg subviral particles in Huh7 cells containing inserts of up to 36 aa from the HCV HVR1 in mammalian cells. The particles were secreted as efficiently as wild-type HBsAg particles (Netter et al., 2001). However, the ability to produce secreted particles is influenced by the way in which the “a” determinant is altered by the insertion of foreign sequences and in particular cysteine rich sequences which dramatically reduced particle secretion (Cheong et al., 2009). Also, immunization of mice with HBs/ HCV genotype 1a or 1b particles resulted in the production of specific antibody responses. Immunization with a combination of these particles resulted in a synergistic effect with stronger antibody responses against both the HVR1 epitopes than with the individual particles (Netter et al., 2001). To determine if pre-existing anti-HBs might reduce the antibody response to the HCV HVR1 sequences included in the chimeric particles these investigators also immunized mice with a commercial HBV vaccine followed by the chimeric HBsAg/HVR1 particles. The presence of pre-existing anti-HBs antibody had no effect on anti-HVR1 antibody titres, suggesting the HBsAg/HVR1 particles could be used in HBV vaccinated individuals (Netter et al., 2001). In addition, anti-HVR1 antisera blocked the entry of HCVpps containing homologous or heterologous HVR1 sequences into Huh7 cells, showing that the chimeric particles were able to induce the production of HVR1-specific neutralizing antibody (Vietheer et al., 2007).

## Retroviral HCV-like particles

Retroviral expression vectors have also proven useful in developing produce neutralizing HCV vaccines. Bellier and coworkers have developed a DNA vaccine expressing murine leukemia virus (MLV) Gag and modified MLV Env proteins capable of assembling into “retroVLPs.” The aim of these particles was to induce stronger immune responses than DNA vaccines expressing single or multiple monomeric proteins. These investigators also developed “plasmo-retroVLPs,” consisting of DNA plasmids that could be used to transform cells to produce retroVLPs carrying foreign epitopes like the E1E2 proteins of HCV (Bartosch et al., 2003; Bellier et al., 2006). RetroVLPs presenting foreign antigens induced stronger immune responses than matching antigens not presented as VLPs (Bellier et al., 2006). RetroVLPs pseudotyped with the Gag protein of MLV and the envelope proteins of Vesicular Stomatitis virus (VSV) or West Nile virus (WNV) were also produced. Antibodies against VSV or WNV were detected earlier and had stronger neutralizing activity than with plasmids not generating VLPs (Bellier et al., 2009). The advantage of this system is that the plasmo-retroVLPs can be combined with the retroVLPs in a prime boost strategy.

These initial studies were followed by a prime-boost immunization series using plasmo-retroVLPs and a recombinant serotype 5 adenovirus (rAd5) expressing HCV-E1/E2 envelope glycoprotein (plasmo-retroVLP^E1E2^) (Desjardins et al., 2009). Mice were primed with rAdE1E2 followed by boosting with plasmo-retroVLP^E1E2^. The plasmo-retroVLP^E1E2^ significantly increased E1E2-specific T cell responses and also increased the production of E2-specific antibody. In comparison, plasmids that were unable to form E1E2-pseudotyped retroVLPs did not produce boosted immune (Desjardins et al., 2009). This was a significant finding as it highlighted the importance of including the HCV E1 and E2 proteins as heterodimers in a particle to ensure that critical neutralizing epitopes are presented correctly.

The rAdE1E2 and plasmo-retroVLP^E1E2^ were next tested in a prime-boost strategy in mice and macaques (Garrone et al., 2011). Immunization of mice with E1E2-expressing vectors elicited anti-E2 antibodies, and the response was significantly enhanced after boosting with retroVLP^E1E2^. A group of four macaques were also primed with rAdE1E2 followed by boosting with retroVLP^E1E2^. Priming with rAdE1E2 resulted in strong T cell responses, but weak anti-E2 antibody responses. In contrast, strong NAb responses were observed after boosting with retroVLP^E1E2^. In addition, the prime boost strategy was shown to produce HCV NAb that cross-neutralized five other HCV genotypes (Garrone et al., 2011). These results highlight the importance of delivering E1E2 on a particulate structure.

In a subsequent study, mice were immunized with plasmo-retroVLPs expressing genotype 1a HCV-envelope glycoproteins and cross-reactive HCV immune responses were assessed by restimulating T cells with heterologous E1E2 antigens from genotypes 1b, 2b, 3a, 4c or 5 (Huret et al., 2013). Stronger T cell responses were found after restimulating with HCV genotype 1b, 3a, and 4c E1E2 VLPs than restimulation with homologous VLPs. To broaden the cross-reactive T cell responses retroVLPs displaying the NS3 polypeptide fused to Gag were produced. Mice were immunized with plasmo-retroVLP^NS3^ and plasmid DNA encoding GagNS3 and the HCV E1E2 proteins. Strong HCV NS3 and E1E2 specific T cell responses were produced. In addition, strong anti-E1 and anti-E2 NAb responses were produced after priming with plasmo-retroVLPs followed by boosting with retroVLP^E1E2^ (Huret et al., 2013).

## Conclusion

Although the development of protective immune responses against HCV is possible, the major challenge facing researchers is how best to reproduce these responses in a vaccine. A HCV VLP-based vaccine has the potential to fulfill the requirements of an effective vaccine for hepatitis C. Several approaches have shown that HCV VLPs are immunogenic and capable of eliciting robust CD4+, CD8+, and cross reactive NAb responses. The results reported with each of these approaches has provided important preclinical data for the progression to broader studies in HCV challenge models and ultimately to clinical trials for development of an effective preventative vaccine for HCV.

## Author contributions

JT wrote the paper in its entirety, created the figures and tables and was responsible for the editing the final draft.

### Conflict of interest statement

The author declares that the research was conducted in the absence of any commercial or financial relationships that could be construed as a potential conflict of interest.
